# Commentary: Is Life Extension Today a Faustian Bargain?

**DOI:** 10.3389/fmed.2018.00073

**Published:** 2018-03-19

**Authors:** Aleksei G. Golubev

**Affiliations:** ^1^N.N. Petrov Research Institute of Oncology, Saint Petersburg, Russia

**Keywords:** aging, mortality, longevity, health, biology

“Such conclusions are always disappointing, but they have the desirable consequences of channeling research in directions that are likely to be fruitful.” Williams G.C. Pleiotropy, natural selection and the evolution of senescence. Evolution. 1957; 11:398–411.

Imagine that in a research field, which flourishes on funds allocated for getting an answer to a pressing question, the answer is eventually found. There will be no need to support the field any further. Specialists who sacrificed their lives to developing it will be uncompetitive in other fields, which are being developed by other scholars. That is, science, unlike practice, needs questions, not answers, which may have value for science only as far as they provoke further questions. In this regard, the value of the commented opinion paper (1) is unquestionable.

Questionable is the practice of extracting quotations out of their full contexts. However, how else can one justify comments on it?

“We’re being offered incrementally smaller amounts of survival time at a very high cost…” (1).

“Smaller” and “very high” are quantitative categories. Is there a way to estimate them by numbers? One way is suggested by the Preston curve, which shows cross-country relationships between per capita gross national product (GNP) and life expectancy (LE) (2). Transforming the plot from its usual appearance, which shows how longevity increases with incomes, into showing the price for increasing longevity (Figure 1), makes it easy to see that increasing the mean age-at-death above ca. 85 years comes at price rocketing to infinity. A similar trick with data about per capita health-care spending will show the same. The hard cold facts reflected by Figure 1 suggest that the results of investing ever-increasing available resources into human life are limited with regard to human life span.

**Figure 1:**
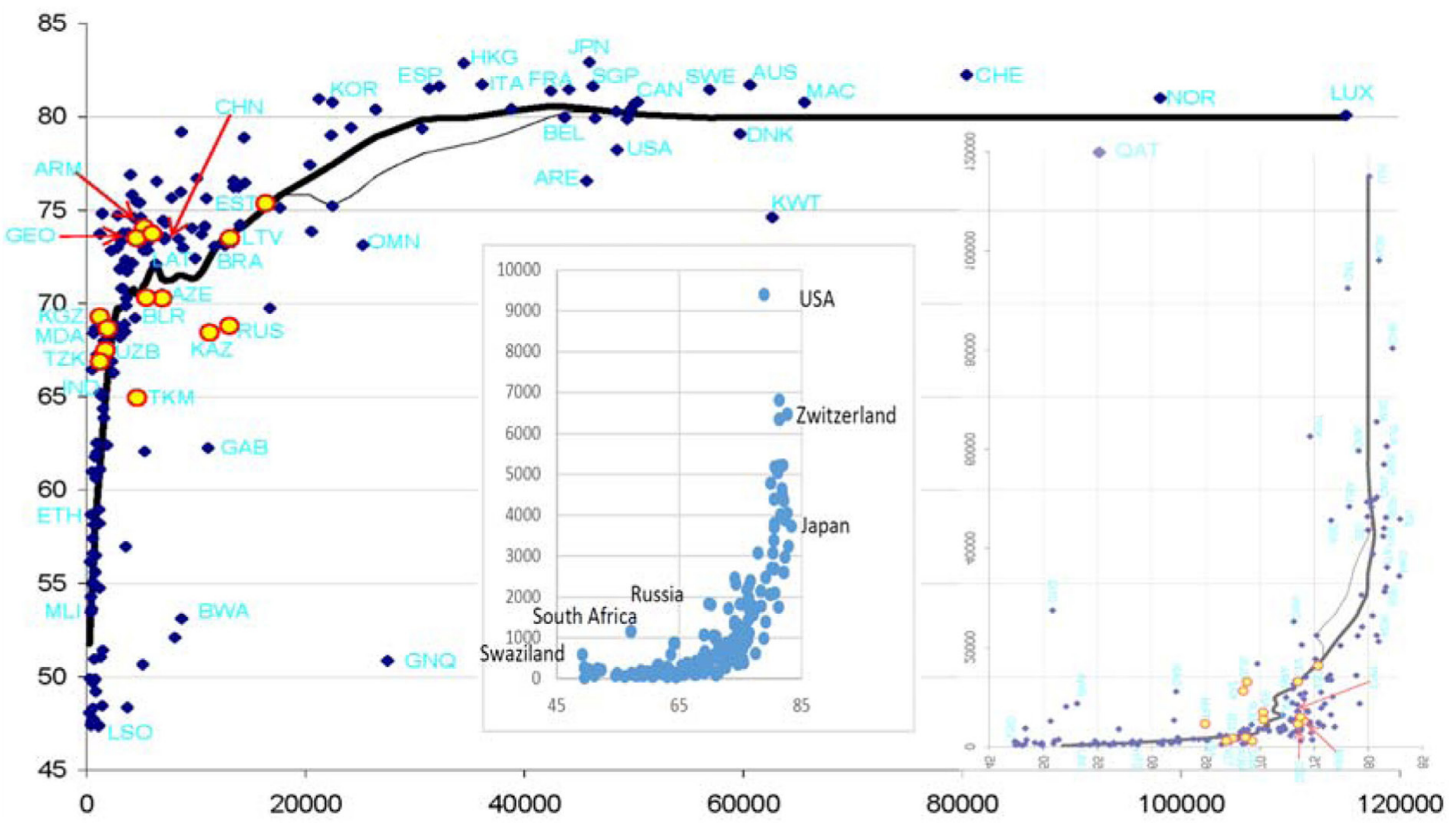
Conventional Preston curve [life expectancy (LE) vs. GDP (in US$)] as of 2010 supplemented with insets showing (right) its transformation into a GDP vs. LE plot and (left) a plot of per capita health-care expenditures vs. LE. The Preston curve is reproduced from Ref. (3). The thick line is obtained by LOESS smoothing. The left inset is based on data available at https://en.wikipedia.org/wiki/List_of_countries_by_total_health_expenditure_per_capita and https://en.wikipedia.org/wiki/List_of_countries_by_life_expectancy.

“A clue about what we should do instead…: … attacking aging itself rather than the diseases associated with it…” (1).

How can one know that aging itself rather than something else is attacked? In populations, aging is manifested as a gradually increasing risk of death with increasing age. This relationship is captured by the Gompertz–Makeham law (GML):

$$\mu \left(t\right)=C+\mu_{0}\times \text{exp}(\gamma \times t),$$

where *μ* captures the probability of death per unit time, *C* is a population-specific parameter, which does not depend on age (*t*), μ_0_ captures the mean initial vulnerability to the causes of death, and *γ* captures the mean rate of the age-dependent increase in vulnerability, i.e., the demographic rate of aging.

Attitudes to GML range from considering it as a manifestation of some natural laws (4) to regarding it as merely a handy tool for describing a current situation (5). The latter attitude implies that the situation can be changed qualitatively without violating any law of nature, provided we can devise a means to do that. The former attitude implies that, because of the exponentially increasing mortality, any finite generation, which overlaps with others to constitute a population, will be inevitably exhausted within a finite time. GML imposes significant constrains on the freedom of thought within the scope of its applicability, as any law does. The respective mortality patterns generate characteristically left-skewed age-at-death distributions and allow calculating GML parameters. Only interventions that influence *γ* may be regarded as targeting “aging itself.” Treating human mortality and survival patterns according to GML suggests that changes in *C* rather than in *γ* are responsible for historical advances in human lifespan (6, 7). Notably, the best ever review on GML and its implications (8) is coauthored by the author of the opinion paper (1) under discussion. Why then GML is not mentioned in the opinion?

“Most important—recent advances in biogerontology suggested that it is plausible to delay aging in people… The Longevity Dividend model seeks to prevent or delay the root causes of disease and disability by attacking the one main risk factor for them all—biological aging” (1).

How can one know that the ability to extend lifespan by influencing aging in nematodes may be expanded to nothing else but aging in humans? In the range from less to more advanced organisms, such as from nematodes through flies to mice, the magnitude of lifespan-modifying effects and their relevance to aging decline, making their projections to human aging uncertain. Rapamycin is an example of this uncertainty (9, 10). Therefore, the relevance of recent advances in experimental life/health span-extending drugs to attacking specifically aging in humans is disputable.

“The modern practitioners of anti-aging medicine try and sell the public what appear to be genuine scientific interventions based on real science, before they’re proven to be safe and efficacious. …” (1).

If paying for anti-aging elixirs offered by anti-aging pharma without due testing is a “Faustian bargain” (which it surely is), how one should esteem testing numerous putative anti-aging drugs for their applicability to humans? Is not it another way of making people pay for the anti-aging agenda?—This time for research (which is supported by taxpayers in the final account) aimed to check whether prospective products are useful, rather than for ready-to-use products having unproved usefulness. Thus, we have another Faustian bargain, albeit more intricate.

Ironically, the most praised “anti-aging” drugs, such as resveratrol, rapamycin, and metformin, are believed to mimic the effects of shifting body energy balance from storage, growth, and self-reproduction to self-maintenance (11, 12). Then what is the reason to use mimetics instead of real things, such as proper calorie intake and adequate physical activity supplemented with moderate alcohol (13–15)? Is it true that the most important bottleneck in increasing health span is the inadequate support of research in anti-aging pharmacology rather than inadequate human attitudes to health? May it be that healthy habits promotion is more cost-effective than anti-aging pills development?

This is not to say that aging research has turned into scholastic exercises performed for their own sake. Delving into the basic mechanisms of aging does help to find novel therapies, which are likely to be overlooked in studies focused on a specific malady. An example is the story of resveratrol, which apparently fails to culminate in a pill to attack human aging, yet continues by patenting new drugs to attack human diseases (16).

## Author Contributions

The author confirms being the sole contributor of this work and approved it for publication.

## Conflict of Interest Statement

The author declares that the research was conducted in the absence of any commercial or financial relationships that could be construed as a potential conflict of interest.
